# Grim19 Attenuates DSS Induced Colitis in an Animal Model

**DOI:** 10.1371/journal.pone.0155853

**Published:** 2016-06-03

**Authors:** Jae-kyung Kim, Seung Hoon Lee, Seon-Young Lee, Eun-Kyung Kim, Jeong-Eun Kwon, Hyeon-Beom Seo, Han Hee Lee, Bo-In Lee, Sung-Hwan Park, Mi-La Cho

**Affiliations:** 1 The Rheumatism Research Center, Catholic Research Institute of Medical Science, The Catholic University of Korea, 222 Banpo-daero, Seocho-gu, Seoul, 137–701, South Korea; 2 Laboratory of Immune Network, Catholic Research Institute of Medical Science, The Catholic University of Korea, 222 Banpo-daero, Seocho-gu, Seoul, 137–701, South Korea; 3 Division of Gastroenterlogy, Department of Internal Medicine, College of Medicine, The Catholic University of Korea, Seoul, Republic of Korea; 4 Division of Rheumatology, Department of Internal Medicine, The Catholic University of Korea, Seoul, 137–040, South Korea; National Institutes of Health, UNITED STATES

## Abstract

DSS induced colitis is a chronic inflammatory disease characterized by inflammation in the gastrointestinal tract, which destabilizes the gut and induces an uncontrolled immune response. Although DSS induced colitis is generally thought to develop as a result of an abnormally active intestinal immune system, its pathogenesis remains unclear. Gene associated with retinoid interferon induced mortality (Grim) 19 is an endogenous specific inhibitor of STAT3, which regulates the expression of proinflammatory cytokines. In this study, we investigated the influence of GRIM19 in a DSS induced colitis mouse model. We hypothesized that Grim19 would ameliorate DSS induced colitis by altering STAT3 activity and intestinal inflammation. Grim19 ameliorated DSS induced colitis severity and protected intestinal tissue. The expression of STAT3 and proinflammatory cytokines such as IL-1β and TNF-α in colon and lymph nodes was decreased significantly by Grim19. Moreover, DSS induced colitis progression in a Grim19 transgenic mouse line was inhibited in association with a reduction in STAT3 and IL-17 expression. These results suggest that Grim19 attenuates DSS induced colitis by suppressing the excessive inflammatory response mediated by STAT3 activation.

## Introduction

DSS induced colitis is a chronic inflammatory disease characterized by dysregulated immune responses in the gastrointestinal tract. DSS induced colitis is an autoimmune disease characterized by chronic and relapsing inflammatory responses in the gastrointestinal tract. As the gastrointestinal tract plays an essential role in the modulation of the immune response to pathogens, unintended weight loss and rectal bleeding [1] make DSS induced colitis a severe inflammatory disorder. To treat DSS induced colitis, downregulation of proinflammatory cytokines, including interleukin (IL)-17, is an important approach [2, 3].

Signal transducer and activator of transcription (STAT) 3, a DNA-binding transcription factor, plays a key role in inflammation by controlling the expression of several cytokines. Indeed, the activation of STAT3 increases IL-17 production [4, 5]. The dysregulated inflammation involved in the pathogenesis of autoimmune diseases is associated with induction of a chronic inflammatory response by STAT3. Autoimmune diseases involve the upregulation of several proinflammatory cytokines, such as IL-17 [6]. Moreover, STAT3 is critical in autoimmune disease—any mutation that induces excessive STAT3 activation initiates several autoimmuned is orders [7]. STAT3 is also involved in the pathogenesis of DSS induced colitis. It has been suggested that haplotype complied by risk alleles of STAT3 polymorphism is significantly related to Crohn’s disease and ulcerative colitis, clinical phenotypes of DSS induced colitis [8]. Indeed, the STAT3 rs744166 ‘A’ allele is a significant risk factor for Crohn’s disease and ulcerative colitis [9]. Furthermore, mucosal T-cells activated by STAT3 contribute to the progression and perpetuation of DSS induced colitis [10]. Thus, STAT3 is an important target in DSS induced colitis therapy. STAT3 inhibition was reported recently to attenuate DSS induced colitis development by suppressing IL-17 production [11].

Gene associated with Retinoid-Interferon-induced Mortality (Grim) 19, generally identified as a nuclear protein, is involved in apoptosis [12]. However, Grim19 is also related to the inflammatory response. Grim19 inhibits nuclear translocation of STAT3 [13] and attenuates experimental autoimmune arthritis by decreasing STAT3 and IL-17 levels [14]. Additionally, overexpression of Grim19 has a therapeutic effect on cancer by reducing STAT3 signaling; in contrast, Grim19 deficiency accelerates tumor development by increasing the expression levels of STAT3-responsive genes [15, 16]. Grim19 production was reduced significantly in the inflamed mucosa of ulcerative colitis [17].

Here, we hypothesized that Grim19 improves DSS induced colitis by ameliorating inflammation. This study aimed to determine whether GRIM19 reduces the expression of STAT3 and proinflammatory cytokines during the colonic inflammatory response in DSS induced colitis, and to explain the underlying mechanism. Thus, we evaluated the therapeutic activity of Grim19 *in vivo* in a mouse model of DSS induced colitis.

## Materials and Methods

### Animals

C57BL/6 mice (Orient, Korea), 8 weeks of age, were maintained under specific pathogen-free conditions at the Institute of Medical Science at the Catholic University of Korea, and were provided standard mouse chow (Ralston Purina, St. Louis, MO, USA) and water *ad libitum*. All experimental procedures were approved by the Animal Research Ethics Committee of the Catholic University of Korea (permit number: CUMC-2015-0185-01), which conforms to all National Institutes of Health of the USA guidelines. To generate GRIM19 transgenic mice, a pcDNA3.1+ (Invitrogen) vector was constructed containing CMV promoter. The GRIM19 fragment was synthesized by GenScript Corporation (NJ, USA), with codon optimization for expression in mammalian cells. The origin of open reading frame is mice. GRIM19 transgenic mice overexpresssing Grim19 were generated on a C57BL/6 background and maintained in facilities at (Macrogen Inc., Seoul, Korea) by microinjection of a transgene. GRIM19 transgenic mice founder transgenic mice were mated to C57BL/6 J mice. The presence of the transgene in the founders was confirmed by polymerase chain reaction (PCR), using genomic DNA extracted from the tail. Mice were euthanized at the end of a study for the purpose of sample collection and histologic examination by CO2 chamber. There are no mice without euthanasia. Mice were monitored daily during experimental term. All surgeries were performed under isoflurane anesthesia with all efforts to minimize suffering. The mice were euthanized for histologic scoring of colon inflammation at pre-determined time points or it they reached the humane endpoints of rectal prolapse, loss of >20% body weight, or signs of pain and distress including poor grooming, decreased activity, and hunched posture. In total, 30 mice were used in this study. Mice were divided into three groups.

### DSS induced colitis induction and injection of Grim19

Colitis was induced exclusively in C57BL/6 mice by oral ingestion of 2.5% dextran sulfate sodium (DSS, MP Biomedicals) for 4 days. To generate a GRIM19 overexpression vector, a *Grim19* fragment was synthesized by TOP gene Technologies (Quebec, Canada) with codon optimization for expression in mammalian cells and sub cloned into the *Bam*HI and *Xho*I sites of pcDNA3.1(+) (Invitrogen). Mice were intravenously injected with Grim19 overexpression vector (100μg/mouse) in 1ml of saline over a 10 speriod, 0 days (100 μg/mice) after DSS induced colitis induction. After 4 and 8 days, the mice received intravenous injection of GRIM-19 overexpression vector. Grim19 Tg mice received 2.5% dextran sulfate sodium (DSS; MP Biomedicals) orally for 4 days. During the periods of DSS and vector injections, animals were inspected daily for body weight.

### Assessment of inflammation

During the experimental period, the severity of colitis was assessed daily by measuring the percentage body weight change and disease activity index (DAI). DAI was calculated as described previously by summarizing the score for body weight loss (0 points, <5% weight loss; 1 point, 5–10% weight loss; 2 points, 10–15% weight loss; 3 points, 15–20% weight loss; and 4 points, >20% weight loss), stool consistency (0 points formed pellets; 2 points, pasty/ semi formed stool; and 4 points, liquid stool) and rectal bleeding (0 points, no rectal bleeding; 2 points, hemoccult-positive; and 4 points, visible gross bleeding).

### Histopathological analysis and immunohistochemistry

Formalin-fixed colon sections were paraffin-embedded and 4μm sections were stained with hematoxylin and eosin (H&E). Assessment included noting of edema, extent of injury, leukocyte infiltration, crypt abscesses, and loss of goblet cells. In this grading system, inflammation severity was scored using a scale of 0–3 (0, no inflammation; 1, slight inflammation; 2, moderate inflammation; and 3, severe inflammation), as the extent of injury (0, no injury; 1, mucosal injury; 2, mucosal and submucosal injury; and 3, transmural injury). Crypt damage was scored using a scale of 0–4 (0, no damage; 1, basal third was damaged; 2, basal two-thirds was damaged; 3, only the surface epithelium was intact; and 4, loss of entire crypt and epithelium). Each value was multiplied by an extent index, ranging from 1–4, that reflected the amount of involvement for each section (1, 0–25%; 2, 26–50%; 3, 51–75%; and 4, 76–100%). Colon tissue were fixed in 4% paraformaldehyde and embedded in paraffin. Then 4μm sections were prepared. Sections were deparaffinized using xylene and dehydrated in a gradient of alcohol solutions. At least four sections from each colon were analyzed. The sections were treated using 1x citrate buffer for antigen retrieval and washed using 1x phosphate buffer saline (PBS, pH7.5). Endogenous peroxidase activity was quenched with methanol and 3% H_2_O_2_, and then blocked with normal goat serum for 30min. Immunohistochemistry (IHC) was performed using the Vecta stain ABC kit (Vector Laboratories, Burlingame, CA). The tissues were first incubated with primary anti-TNF-α, anti-IL-1β, anti-IL-8, anti-IL-17, anti-STAT3, anti-Grim19 (all from Santa Cruz Biotechnology, Santa Cruz, CA, USA), and anti-IL-6 (Abcam) antibodies overnight at 4°C; followed by a biotinylated secondary linking Ab and a streptavidin–peroxidase complex for 1h. The final color product was developed using 3,3-diaminobenzidine, (DAKO, Carpinteria, CA). The sections were counterstained with Mayer’s hematoxylin. All histological assessments were performed by two independent blinded observers. Images were captured using a DP71 digital camera (Olympus, Center Valley, PA) attached to an Olympus BX41 microscope. We counted cell number using Panoramic MIDI and panoramic and viewer (3DHISTECH Ltd, Hungary).

### Confocal microscopy

For immunostaining, 5μm tissue sections of the colon or mesenteric lymph node were stained using fluorescein is othiocyanate (FITC)-conjugated anti-CD4, phycoerythrin (PE)-conjugated anti-IL-17, PE-conjugated anti-*p*-STAT3 Y705, PE-conjugated anti-*p*-STAT3 S727, and anti-Grim19 (SantaCruz) antibodies overnight at 4°C. Tissue sections were washed with PBS and incubated with fluorescence-conjugated secondary goat anti-rabbit IgG. Stained sections were visualizedusing a Zeiss microscope (LSM 510 Meta; Carl Zeiss, Oberkochen, Germany).

### Quantitative polymerase chain reaction (qPCR) analysis of gene expression

Total RNA was extracted using TRIzol reagent (Molecular Research Center. Cincinnati, OH, USA). The RNA concentration in each sample was measured using a NanoDrop ND-1000 (Thermo Fisher Scientific, MA, USA). Total RNA (2 μg) was reverse transcribed into cDNA using the Transcriptor First-Strand cDNA Synthesis Kit (Roche Applied Science). mRNA levels were estimated using qPCR assays with FastStart SYBR Green Master Mix (Roche Applied Science) using StepOnePlus (Applied Biosystems) according to the manufacturer’s instructions. Relative mRNA levels were normalized to that of β-actin. Primer sequences are listed in Table 1.

**Table 1:** PCR primers used in this study.

Gene	Sense Primer(5'-->3')	Anti-sense Primer(3'-->5')
β-actin	GAA ATC GTG CGT GAC ATC AAA G	TGT AGT TTC ATG GAT GCC ACA G
IL-6	ATG CTC CCT GAA TGA TCA CC	TTC TTT GCA AAC AGC ACA GC
IL-17	CCT CAA AGC TCA GCG TGT CC	GAG CTC ACT TTT GCG CCA AG
IL-8	CTA GGC ATC TTC GTC CGT CC	TTC ACC CAT GGA GCA TCA GG
IL-1β	GGA TGA GGA CAT GAG CAC ATT C	GGA AGA CAG GCT TGT GCT CTG A
TNF-α	AAG CCT GTA GCC CAC GTC GTA	GGC ACC ACT AGT TGG TTG TCT TTG
Grim19	GAA GGA TGT GCC CAA CTG GA	GTG TAC CAG GTG AAG CCG AA

### Western blot analysis

HEK293 cells isolated from control or GRIM19 treated mice were washed with cold saline, and total proteins extracted with lysis buffer [1% Nonidet P-40, phenyl methane sulfonyl fluoride (PMSF), 2mM sodium vanadate, 0.1% sodium deoxycholate, and a protease inhibitor mixture; Roche Applied Science, Mannheim, Germany]. Harvested lysates were centrifuged (15min, 4°C to pellet cellular debris. Proteins were loaded onto 10% polyacrylamide gels and subjected to sodium dodecyl sulfate polyacrylamide gel electrophoresis, and then transferred to nitrocellulose membranes (Invitrogen Life Technologies, Carlsbad, CA, USA). Membranes were blocked with 5% (w/v) non-fat milk in Tris-buffered saline with 0.1% Tween-20 (TBST) for 1h at room temper-ature and incubated with antibodies against GRIM19 and β-actin overnight at 4°C. Membranes were then incubated with goat anti-mouse horseradish peroxidase-conjugated antibodies. Immunoreactivity was determined using enhanced chemiluminescence reagents (Amersham Biosciences, Piscataway, JN, USA).

### Statistical analysis

All data are means ± standard deviation (SD). One-way ANOVA with Bonferroni's *post hoc* test for multiple comparisons were used for statistical analysis using Graphpad Prism v.5.01. *P*-values less than 0.05 were considered to indicate statistical significance.

## Results

### Grim19 improved DSS induced colitis

**Fig 1:**
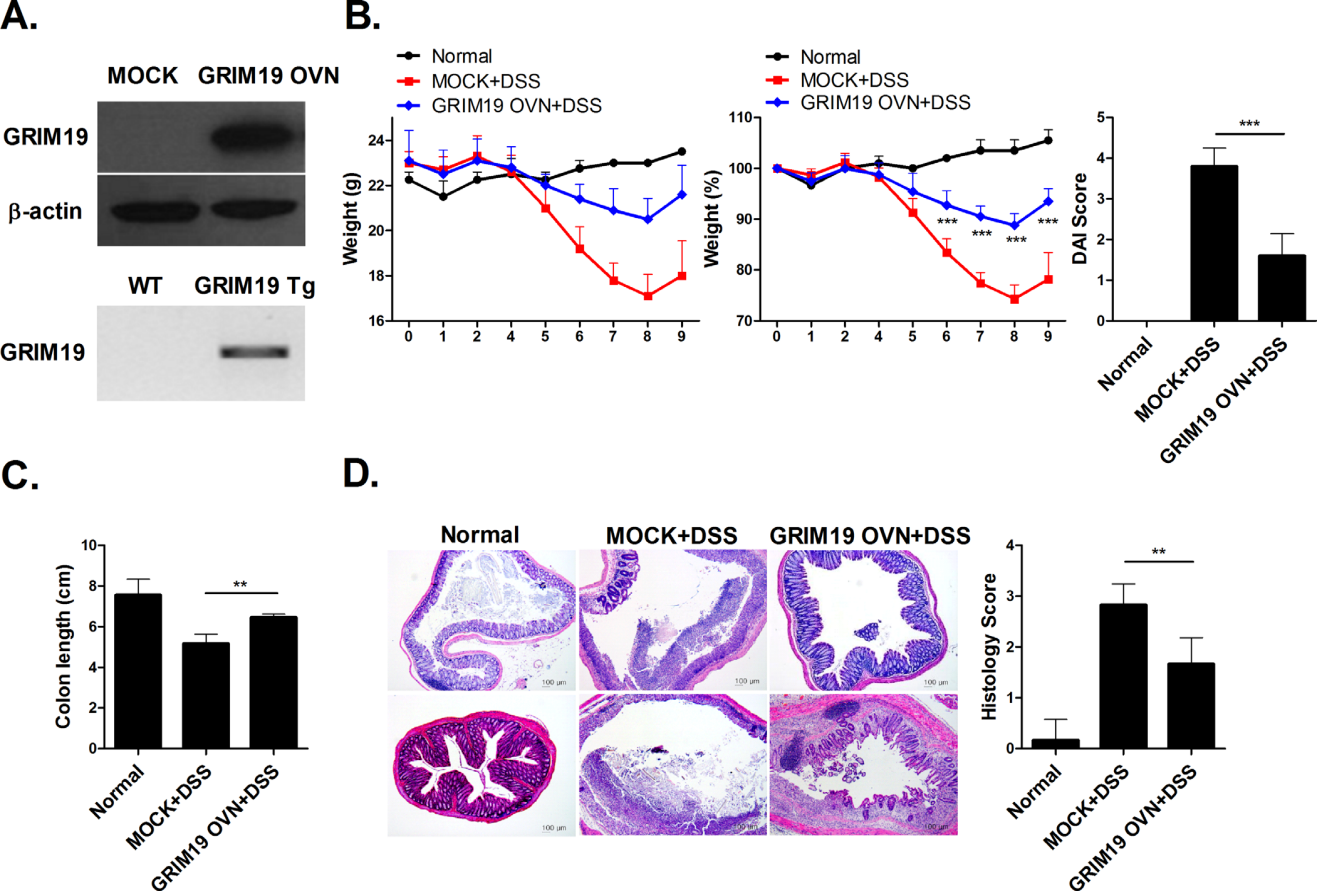
Grim19 prevented induction of DSS induced colitis. (A) Mock or GRIM19 overexpression vectors were administered to HEK293 cell and GRIM19 transgenic mice overexpresssing Grim19 were generated on a C57BL/6 background. Lystes of Mock vector or GRIM19 vector transfected HEK293 cells were analyzed for GRIM19 protein expression using western blotting with and anti-GRIM19 antibody. Transgene expression were detected by PCR products. (B) Change in body weight during the disease process. The DAI score in mice with DSS induced colitis decreased significantly. (n = 5) (C) Colon length. (n = 5) (D) Histopathological changes in the colon. Sections were stained with hematoxylin and eosin (H&E). Scale bar = 100um Values are means±SD of three independent experiments.(n = 5) **P<0.01, ***P<0.001.

As Grim19 attenuates autoimmune disease [14], we determined whether Grim19 exerted a therapeutic effect in DSS induced colitis. Mock or GRIM19 overexpression vectors were administered to HEK293 cells. GRIM19 transgenic mice overexpresssing Grim19 were generated on a C57BL/6 background. We found that GRIM19 overexpression vectors results in upregulation of GRIM19 level and transgene expression in C57BL/6. (Fig 1A). Grim19 treatment improved DSS induced colitis in mice in terms of reducing DAI scores, body weight (Fig 1B) and colon length (Fig 1C). Moreover, Grim19 treatment suppressed colon inflammation in DSS induced colitis mice (Fig 1D).

### Grim19 decreases the expression of proinflammatory cytokines in the DSS induced colitis colon

**Fig 2:**
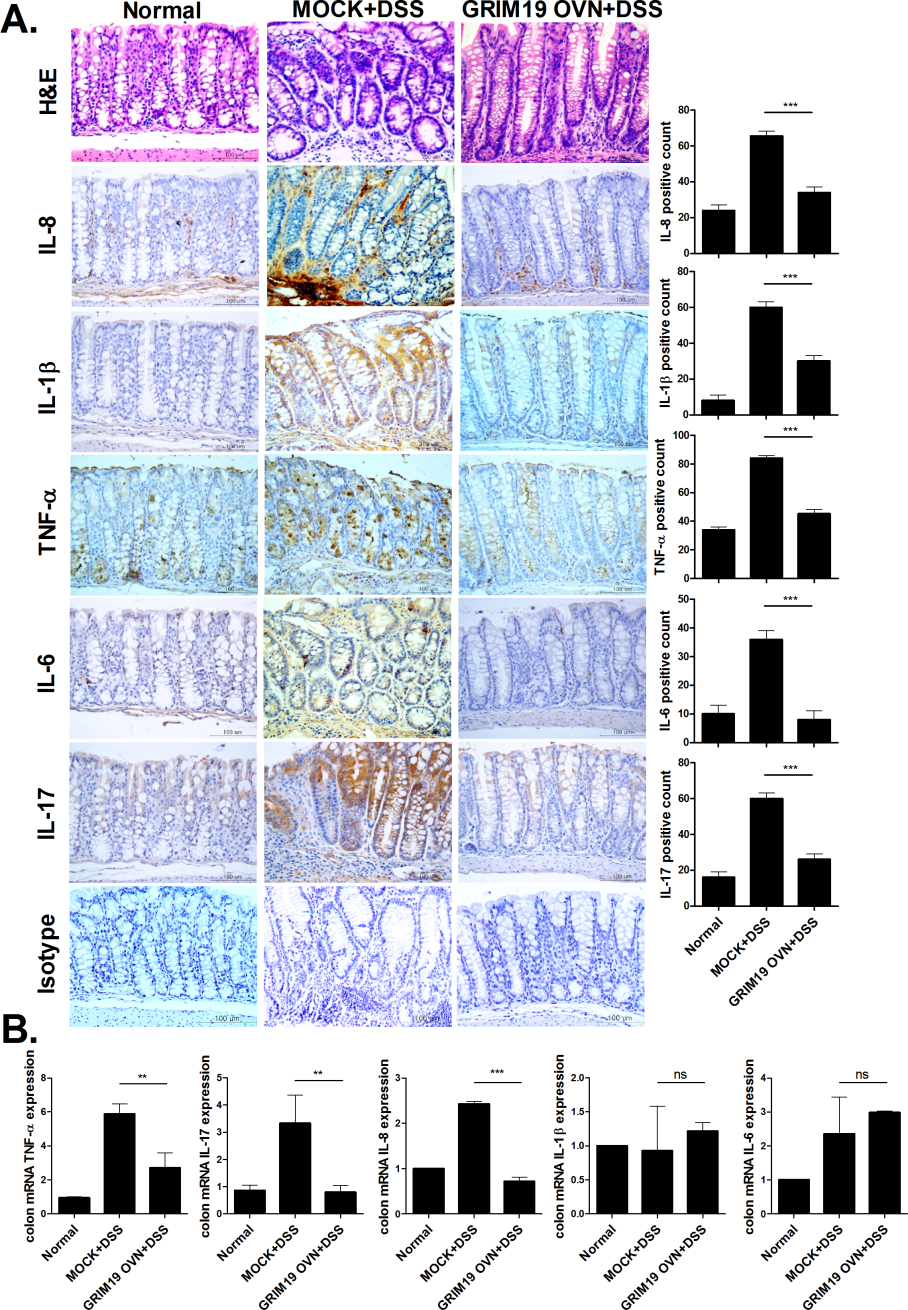
GRIM19 controls the expression of proinflammatory cytokines in the DSS induced colitis colon. (A) H&E and immunohistochemical staining of IL-8, IL-1β, TNF-α, IL-6 and IL-17 in colon tissue of mice with DSS induced colitis and mice with DSS induced colitis that received GRIM19 injection. Scale bar = 100um (n = 5) (B) Expression of IL-17, IL-6, IL-8, IL-1β and TNF- α genes in the colon was measured by real-time polymerase chain reaction (n = 5). Values are means ±SD of three independent experiments. **P<0.01, ***P<0.001.

To assess the therapeutic effect of Grim19 on colonic inflammation, we performed immunohistochemical staining of proinflammatory cytokines. The IL-1β, -6, -8, -17 and TNF-α protein levels were downregulated significantly in DSS induced colitis mice treated with Grim19 compared with control mice (Fig 2A). Further, relative TNF-α, IL-17, IL-6, IL-8 and IL-1β mRNA levels in colon tissue were decreased significantly by Grim19 treatment (Fig 2B).

### Grim19 decreases STAT3 expression in the DSS induced colitis colon

**Fig 3:**
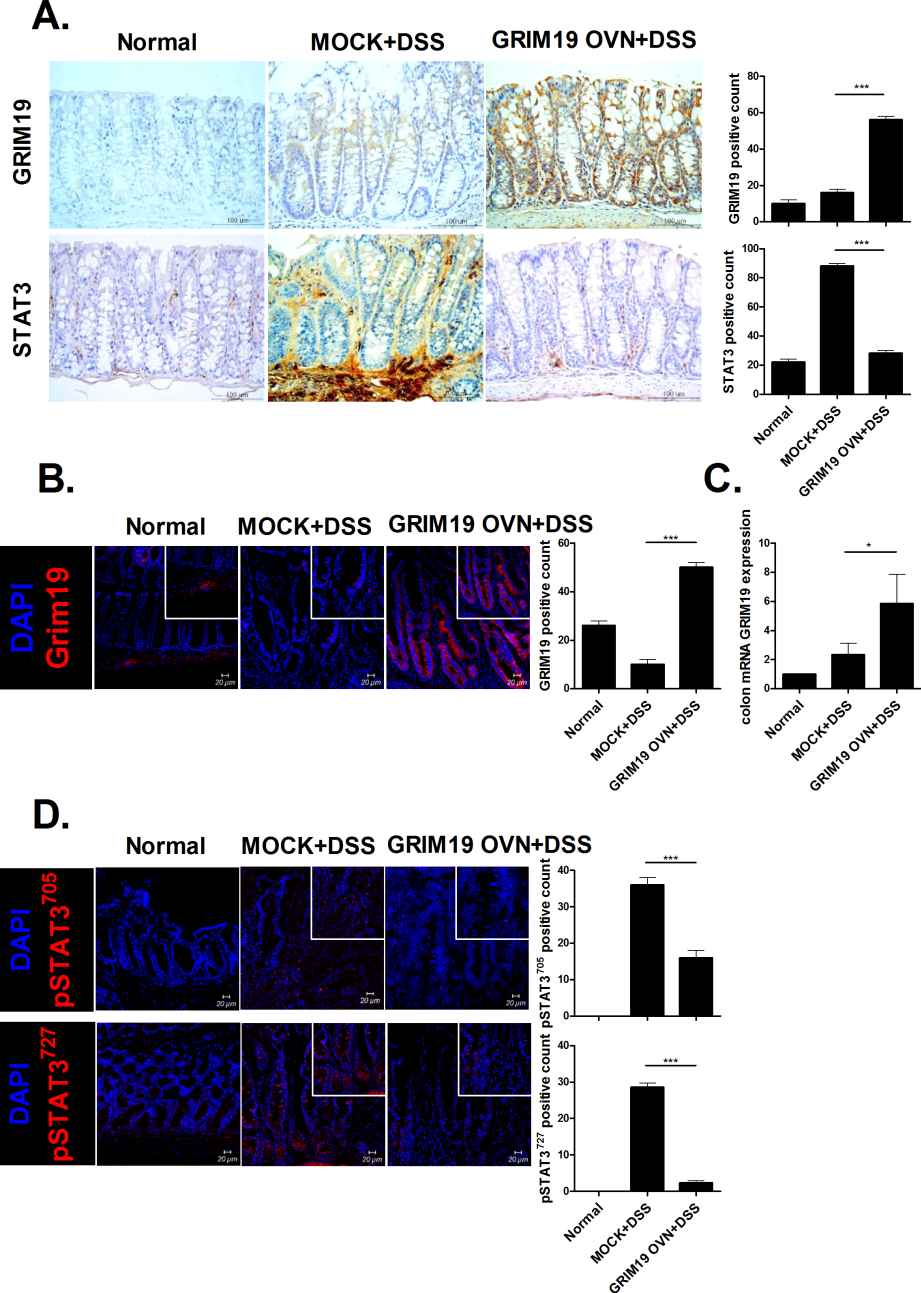
Grim19 suppresses inflammation and p-STAT3 expression. (A) Immunohistochemical staining of Grim19 and STAT3 in colon tissue. (n = 3) (B) Immunofluorescence staining of Grim19 colon tissue. (n = 3) (C) Grim19 expression was determined by real-time polymerase chain reaction. (n = 5) (D) Colons of Grim19-injected mice with DSS induced colitis and mice with DSS induced colitis (controls) were subjected to immunostaining for pSTAT3^727^ and pSTAT3^705^ (n = 3). Scale bar = 100um, 20um Values are means±SD of three independent experiments. *P<0.05, ***P<0.001.

Grim19 expression was increased significantly, whereas STAT3 production was reduced significantly, in DSS induced colitis mice treated with Grim19 compared with control mice (Fig 3A). Moreover, Grim19 expression was significantly induced in DSS induced colitis mice treated with Grim19 compared with control mice (Fig 3A and 3B). However, p-STAT3^705^ and ^727^expression was decreased significantly in DSS induced colitis mice treated with Grim19 compared with control mice (Fig 3D).

### Grim19 decreases STAT3 expression in the lymph node in DSS induced colitis

**Fig 4:**
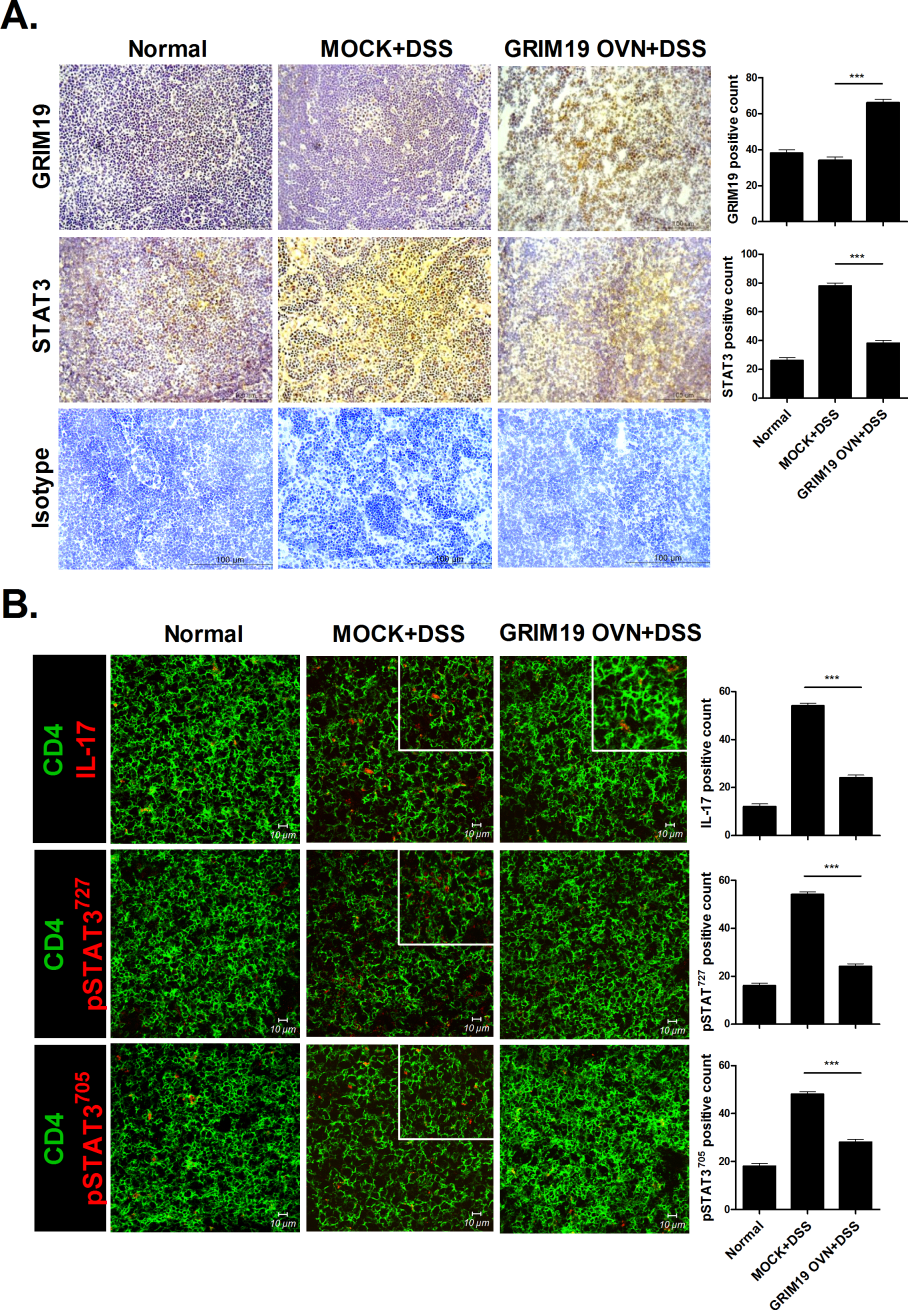
Grim19 suppresses inflammation and p-STAT3 expression. (A) Immunohistochemical staining of Grim19 and STAT3 in lymph node tissue (n = 3). (B) Immunofluorescence staining of lymph node tissues (n = 3). Scale bar = 100um, 10um Values are means±SD of three independent experiments. ***P<0.001.

As the mesenteric lymph node is important in DSS induced colitis progression [18], we determined Grim19, STAT3 and IL-17 expression in the mesenteric lymph nodes of mice with DSS induced colitis. Grim19 production was increased significantly, whereas STAT3 expression was decreased significantly, in DSS induced colitis mice treated with Grim19 compared with control mice (Fig 4A). Confocal microscopy showed that IL-17, p-STAT3^705^ and ^727^expressionwas reduced significantly in DSS induced colitis mice treated with Grim19 compared with control mice (Fig 4B).

### Improvement of DSS induced colitis in GRIM19 transgenic mice

**Fig 5:**
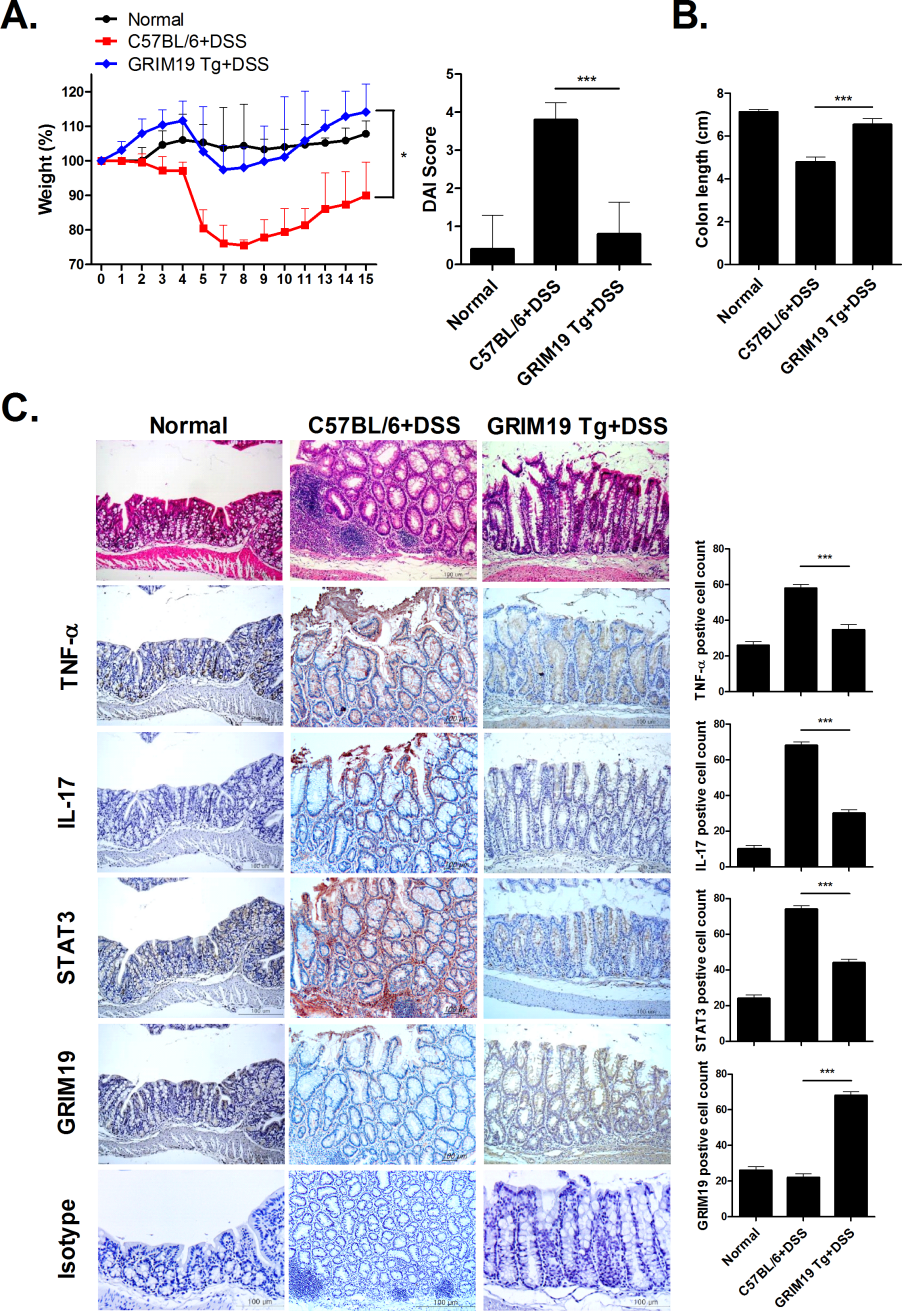
Attenuation of DSS induced colitis in Grim19Tg mice. (A) Change in body weight during the disease course. The DAI score in mice with DSS induced colitis was significantly lower than that in Grim19 Tg mice with DSS induced colitis (n = 5). (B) Colon length (n = 5). (C) H&E and immunohistochemical staining of TNF-α, IL-17, STAT3 and GRIM19 in colon tissue of mice with DSS induced colitis and GRIM19-Tg mice with DSS induced colitis (n = 3). Scale bar = 100um Values are means±SD of three independent experiments. *P<0.05, ***P<0.001.

To confirm the therapeutic effect of Grim19, we induced DSS induced colitis in WT and Grim19 Tg mice. Weight loss and DAI scores were significantly decreased in Grim19 Tg mice compared with control mice (Fig 5A). However, colon length was maintained in Grim19 Tg mice (Fig 5B). Grim19 Tg mice also exhibited protection of colon tissue against DSS induced colitis associated degradation and decreased proinflammatory cytokine protein levels in colon tissue, but increased Grim19 expression (Fig 5C).

## Discussion

Grim19 has been primarily investigated in apoptosis and cancer because it diminishes STAT3 activation [15, 19]. However, Grim19 is also associated with inflammatory diseases. Grim19 exerted an anti-arthritic effect by reducing the activation of STAT3 [14]. Moreover, Grim19 expression was reduced in inflamed mucosa of patients with intestinal inflammatory disease [17]. However, there is little evidence of its therapeutic function with respect to the inflammatory response in DSS induced colitis. Although Grim19 regulates the responses of intestinal epithelial cell to microbes [17], no report of the therapeutic activity of Grim19 through inhibition of p-STAT3 has been published. To our knowledge, this is the first study to report the therapeutic effect of Grim19 in DSS induced colitis. We report that Grim19 reduces DSS induced colitis development, which will facilitate DSS induced colitis therapy.

The therapeutic function of Grim19 in DSS induced colitis is a novel finding. The therapeutic activity of Grim19 resulted in inhibition of p-STAT3 expression. STAT3 plays an important role in the pathogenesis of DSS induced colitis. p-STAT3 promoted the expression of proinflammatory cytokines, including TNF-α and IL-1β, in patients with intestinal inflammatory disease [20, 21]. The transcriptional activity of STAT3 has been suggested to induce TNF-α production and plays a role in the pathogenesis of DSS induced colitis [22]. Thus, inhibition of STAT3 attenuates DSS induced colitis severity by reducing proinflammatory cytokine levels during inflammation of the colon [11]. Moreover, the IL-6/STAT3 pathway is an important target in DSS induced colitis therapy [10]. Transgenic mice overexpressing Grim19 exhibited improvement of DSS induced colitis progression due to downregulation of p-STAT3 and proinflammatory cytokines in the colon. Our results suggest that Grim19 inhibits proinflammatory cytokine and p-STAT3 expression to suppress inflammation, which suggests a novel therapeutic strategy for DSS induced colitis.

At present, several immunosuppressive drugs for DSS induced colitis have been developed. For example, azathioprine, 6-mercaptopurine, methotrexate, cyclosporine and tacrolimus can be used in DSS induced colitis as immunosuppressives [23]. However, cyclosporine has several side effects, such as renal dysfunction, hypertension, vomiting, diarrhea and peptic ulcer [24]. Azathioprine and 6-mercaptopurine are ineffective in 33% of patients with intestinal inflammatory disease; moreover, up to 20% of these patients cease taking these drugs due to adverse effects [23]. Moreover, many DSS induced colitis drugs, including TNF-α blockers, are associated with opportunistic infections, malignancies and autoimmunity [23]. Thus, novel drugs for DSS induced colitis that lack side effects are needed.

A Janus kinase inhibitor has been used in DSS induced colitis. Tofacitinib, an oral JAK inhibitor, was used in DSS induced colitis therapy and showed promising efficacy [25]. Since STAT3 is inhibited by oral JAK inhibitors [26], STAT3 inhibitors may also be useful for DSS induced colitis treatment. Indeed, metformin exhibited a therapeutic effect by decreasing STAT3 activation in mice with DSS induced colitis [11]. Grim19 is a STAT3 inhibitor and therapeutic for autoimmune arthritis [14, 15]. Therefore, Grim19 maybe a good candidate agent for DSS induced colitis treatment.

STAT3 is essential transcription factor in normal state whereas, in inflammatory condition, STAT3 can be a severe pathogenic transcription factor [11, 14]. In this study, we hypothesized that STAT3 inhibition can reduce colitis severity because colitis is chronic inflammatory disease and observed that STAT3 inhibition mediated by Grim19 improved acute colitis. But, STAT3 reveals an opposing function such as double edge sword in colitis. There are several evidences that STAT3 deletion can induce severe colitis progress and DSS-induced intestinal injury [27–29]. We cannot exclude the possible adverse effects of STAT3 inhibition in long term because this investigation is confined to in vivo tests during short term. However, this study is the first to report the function of Grim19 in the inflammatory response of colitis. *In vivo* animal research are required to further validate our hypothesis prior to the clinical trial of Grim19 in intestinal inflammation.

## Conclusion

Our results suggest a therapeutic effect of Grim19 in intestinal inflammation. The therapeutic activity of Grim19 and its ability to inhibit STAT3 activation have been reported previously [14]. In this study, Grim19 inhibited DSS induced colitis progression and weight loss in mice by downregulating the production of STAT3 and proinflammatory cytokines, including TNF-α and IL-6, in colonic inflammation. These results suggest that Grim19 can ameliorate DSS induced colitis by inhibiting STAT3. Therefore, Grim19 may be a good candidate agent DSS induced colitis treatment.
